# Mating, but Not Male Accessory Gland Products, Changes Female Response to Olfactory Cues in *Anastrepha* Fruit Flies

**DOI:** 10.3389/fphys.2021.714247

**Published:** 2021-09-09

**Authors:** Guadalupe Córdova-García, Laura Sirot, Solana Abraham, Francisco Díaz-Fleischer, Norma Flores-Estevez, Maurilio López-Ortega, Diana Pérez-Staples

**Affiliations:** ^1^INBIOTECA, Universidad Veracruzana, Xalapa, México; ^2^Department of Biology, College of Wooster, Wooster, OH, United States; ^3^Laboratorio de Investigaciones Ecoetológicas de Moscas de la Fruta y sus Enemigos Naturales (LIEMEN), PROIMI-Biotecnología, CONICET, San Miguel de Tucumán, Argentina

**Keywords:** accessory glands, Diptera, pheromone, olfactometry, volatiles, testectomy, Tephritidae, ejaculate

## Abstract

Copulation and/or ejaculate components can alter female physiological state and female post-mating behavior. The objective of the present study was to determine if copulation and male reproductive accessory gland products (MAGs) modify the behavior of female *Anastrepha ludens* (Loew) and *Anastrepha obliqua* (Macquart; Diptera: Tephritidae) in response to two stimuli: male-emitted pheromone and oviposition host volatiles. Olfactometry studies revealed that mated females of both *A. ludens* and *A. obliqua* have a stronger response for host volatiles compared to unmated females, which have a stronger response for male pheromone. We also examined olfactory responses of females mated to testectomized males who could transfer MAGs but not sperm. In both species, MAGs alone did not cause the change in the olfactory response observed after copulation, unlike what has been found in *Ceratitis capitata* (Wiedemann). Females mated to testectomized males responded equally to the male sex pheromone or to host volatiles, thus suggesting that the whole ejaculate is needed to elicit the complete behavioral switch in olfactory response. The function of MAGs is still unknown in these two pests of economic importance. The response for host volatiles by mated females has implications for the development of baits and traps that should preferably attract and target this population.

## Introduction

In insect reproduction, the stimuli that females receive during copulation can produce a series of morphological and physiological changes. For example, they can cause a higher production and release of eggs (Judson, 1967; Loher and Edson, 1973; Yu et al., 2014), change in food intake (Judson, 1967; Carvalho et al., 2006; Villarreal et al., 2018), an increase in the secretion of excreta (Cognigni et al., 2011; Apger-McGlaughon and Wolfner, 2013), changes in the reproductive system (Heifetz and Wolfner, 2004; Avila et al., 2011; Heifetz et al., 2014) and digestive system (White et al., 2021), formation of genital plugs (Takami et al., 2008), change in lifespan and immune response (Lung et al., 2001; Yamane and Miyatake, 2010; Short et al., 2012; Yu et al., 2014; Croshaw and Gómez, 2018) and decreased sex pheromone production (Kingan et al., 1993). These changes can occur mainly due to two stimuli produced during copulation: ejaculate transfer and mechanical factors.

The ejaculate is composed of sperm, male accessory gland products (MAGs; Gillott, 2003; Perry et al., 2013), and secretions produced by the ejaculatory bulb and ducts (Chapman, 2001; Avila et al., 2011). Transfer of sperm can trigger oocyte and embryonic development (Avila et al., 2010; Findlay et al., 2014; Wainwright et al., 2021), whereas the mode of action of MAGs is not known for most species, in some species, these stimuli act on the female nervous system, causing the release of neurosecretions that generate changes in the female (Engelman, 1970; Heifetz and Wolfner, 2004; Yapici et al., 2008; Avila et al., 2012; Rubinstein and Wolfner, 2013; Heifetz et al., 2014). These changes in female postcopulatory phenotypes are particularly relevant for insect pests, as this information may allow us to develop tools for their successful control.

In tephritid flies (Diptera: Tephritidae), copulation produces a reduction in female response to male sex pheromone (Robacker et al., 1985; Devescovi et al., 2021) and MAGs specifically, can decrease female receptivity (Radhakrishnan and Taylor, 2007; Abraham et al., 2012). In the Mediterranean fruit fly *Ceratitis capitata* (Wiedemann), apart from inhibiting sexual receptivity (Jang et al., 1999), MAGs can also change the olfactory response of unmated females from a response to the male sex pheromone to a higher attraction to oviposition host volatiles (Jang, 1995). However, it is not known if MAGs have the same effect in other tephritids. Thus, we aimed to evaluate if copulation and MAGs change the olfactory behavior of females of two tephtritid species, the Mexican fruit fly *Anastrepha ludens* (Loew), which mainly infests citrus fruits, and the West Indian fruit fly *Anastrepha obliqua* (Macquart), which mainly infests mangoes and plums (Hernández-Ortiz and Aluja, 1993). Studying the response of reproductively mature females to their host has implications for bait and trap development for the control of fruit flies of economic importance.

*Anastrepha ludens* and *A. obliqua* exhibit different characteristics during and after copulation. For example, *A. ludens* only copulates at dusk, whereas *A. obliqua* copulates throughout the day, showing a preference peak during the morning. Copulation in *A. ludens* lasts a mean of 73.4 (±6.6) minutes, whereas in *A. obliqua* it lasts a mean of 47.1 (±0.9) minutes (Aluja et al., 2000). The mechanical stimulus of copulation is enough to induce an increase in number of eggs laid in *A. ludens*, even when females do not receive an ejaculate when copulating with males with a severed distiphallus (Reyes-Hernández et al., 2021). Furthermore, copulation with all the stimuli in *A. ludens* causes a higher female response to host volatiles (Robacker and Garcia, 1990), while in *A. obliqua* copulation does not have that effect (López-Ley et al., 2016). For both species, females usually remate. The mean sexual refractory period in *A. ludens* is 12days and in *A. obliqua* is 17days (Aluja et al., 2009). A reduction in the response to male pheromone after copulation has been reported for *A. ludens*, but we do not yet know what stimuli influences this behavioral change (Robacker et al., 1985).

The function of MAGs in both *A. ludens* and *A. obliqua* is still unknown. In *A. ludens*, MAGs do not have an effect on mating inhibition, oviposition, or ovary size (Abraham et al., 2014, 2016; Reyes-Hernández et al., 2021). In *A. obliqua*, it is only known that females become receptive faster after copulating with a male with a depleted ejaculate (Pérez-Staples and Aluja, 2006), and this phenomenon has been attributed to depletion of MAGs and not of sperm (Pérez-Staples et al., 2008). Therefore, to address the gap in knowledge about the role of MAGs in these species, we determined the effect of copulation and MAGs on the behavior of *A. ludens* and *A. obliqua* females in response to two stimuli: male-emitted pheromone and host volatiles. Based on findings from other tephritids (Jang, 1995), we predicted that unmated females would respond to male pheromone over host volatiles when compared with mated females, and that the stimulus for this behavioral change would be through the receipt of MAGs.

## Materials and Methods

### Insects and Rearing

We collected *A. ludens* from infested sour oranges (*Citrus aurantium* L.) and grapefruit (*Citrus paradasi* L.) and *A. obliqua* from fruits of hog plum (*Spondias mombin* L.), purple mombin (*S. purpurea* L.), and rose apple (*Syzygium jambos* L. Alston) in the localities of Tuzamapan, La Estanzuela, Vega de la Torre and El Roble, Veracruz, Mexico. The flies obtained from the wild fruits were presented with mangoes in which to oviposit. For our experiments we used flies from the first eight generations for *A. ludens* and from the first three generations for *A. obliqua* both reared in mango. The fruits were placed in containers with vermiculite or soil to allow larvae to emerge and pupate. After emergence, adult flies were sorted by sex in cages with tulle mesh (30cm×30cm×30cm). Adults were fed with a mixture of sugar and hydrolyzed protein in a proportion of 3:1 (MP Biomedicals, LLC, Santa Ana, California, United States) and were provided with water in a container with moist cotton. Laboratory conditions were: 25±1°C, 60±10% RH, and a 12:12h light–dark photoperiod.

### Copulations

We used flies of 15–30 days of age for *A. ludens* and 15–23days for *A. obliqua*, as at this age they are sexually mature (Aluja et al., 1983; Reyes-Hernández and Pérez-Staples, 2017). One day prior to olfactometer experiments (described below), we placed females and males in an observation cage in a ratio of 1:1 without food and water. The number of flies in a cage varied depending on the number available range: 20–80 pairs of flies. Only copulations that lasted more than 15min were used. We provided foliage in the form of a small branch of *Citrus* for *A. ludens* and of *S. purpurea* for *A. obliqua* placed inside a 150ml jar. Wild flies have been shown to have increased mating success when provided foliage as a stimulus (Díaz-Fleischer et al., 2009). Foliage was replaced daily. Copulas were observed for *A. ludens* from 15:00 pm to 20:00 pm and for *A. obliqua* from 7:00 am to 15:00 pm. Pairs were carefully taken out of the cage while mating occurred and at the end of copulation the males were discarded. Mated females were placed in a cage with food and water to be observed in the olfactometer 24h after mating. Individuals that did not copulate were discarded. Unmated females were from a separate cage which had no contact with males. Unmated and mated females were used for the olfactometry experiments.

### Olfactometry Experiments

Olfactometry experiments were conducted at the Instituto de Biotecnología y Ecología Aplicada (INBIOTECA) of the Universidad Veracruzana. We used a Y-shaped glass olfactometer, where females could not see the fruit or the males and could only perceive chemical cues. The air flow inside the Y-tube was generated with an air pump (Cole Parmer Model No. L-79 200-00) connected to the end of the device. The air entered the system through a pair of flowmeters (Cole Parmer). The flow rate was set at 0.2L min. Briefly, incoming air was passed through an activated charcoal filter, and humidified by passing it through a jar with distilled water (300ml). The air current entered two glass jars (900ml) containing the odor source. The two jars were connected to one of the arms of the Y-tube. The Y-tube comprised of two arms that were 8cm long that converged in a central tube that was 25cm long. In the central tube, the odours mixed. There was an 80° angle between the arms, and the glass tubes had a diameter of 65mm. The end of the central tube had an orifice through which the flies were introduced. All the parts were connected using colorless and odorless hoses (plasticizer-free, translucent, and with chemical compatibility to weak and medium acids and bases, salts and alcohols. Tubing Cflex® 3/16×5/16 25’ Cole Parmer). The Y-tube was illuminated with a cold white light lamp (400 lm) that provided homogeneous illumination of 1,200±100 lux. The room was kept at 26±1°C and 60±10% relative humidity. These conditions were only maintained when the tests were carried out. Before and after testing flies were returned to the rearing laboratory.

### Effect of Copulation on Response to Male Sex Pheromone or Host Volatiles

One day after females were observed copulating, they were tested along with unmated females for their olfactory response. We activated the olfactometer air pump 3min before starting the trials to fill the olfactometer with test odours. We then placed three females (unmated or mated) in the entrance of the olfactometer and gave them 30min to choose one of the arms. We placed three females based on a previous unpublished study and preliminary tests.

#### Pheromone or Host Versus No Stimulus

To have a baseline for activity, females were given the choice between one jar with 10 sexually mature males of the same age as the females, wing-fanning and emitting pheromone, and one empty jar (no stimulus; *N*=45 *A. ludens* and *N*=54 *A. obliqua*). In a separate experiment, females were given the choice between one jar with host fruit and one empty jar. For *A. ludens*, one orange was used as a host and for *A. obliqua*, 10 unripe hog plums or plums (*Spondias* spp.; 65±25gr) were used as host stimuli. Considering that *A. ludens* oviposits in the morning but mates in the evening, we carried out experiments with host volatiles vs. no stimulus both in the morning from 9:00 to 15:00h (*N*=55), and the afternoon from 15:00 to 19:00h (*N*=36). For *A. obliqua* experiments were carried out in the morning (*N*=62).

#### Pheromone Versus Host

Females were given the choice between one jar with 10 sexually mature males of the same age as the females, wing-fanning and emitting pheromone, and one jar with host fruit. We used one unripe orange (*C. aurantium*; 80±25gr) with five scratches and two leaves (each leaf was cut into five pieces) for *A. ludens* (*N*=56), and 10 unripe plums for *A. obliqua* (*N*=44).

We considered that a female had made a choice when she entered one of the arms, exceeded a distance of 4cm from the end of the central tube, and remained beyond that distance for 3min. We recorded the chosen option for a single female. If the females did not choose an option after the 30min, the trial was concluded, and the next set of females were arbitrarily chosen. After each trial, we switched to a clean Y-tube (cleaned with 96% ethanol). The position of the jars with the odor sources was rotated arbitrarily to avoid side bias. Each female was used only once. After each day of observation, all Y-tubes and glass jars were rinsed with hot water (78°C) and then cleaned with 96% ethanol. The experiment was conducted blind, that is, the observer did not know the identity of the female (unmated or mated).

### Effect of Accessory Gland Products on Response to Male Sex Pheromone and Host Volatiles

#### Testectomy

In order to remove the testes, we performed a microsurgery under a stereo microscope (Olympus SZX7, Tokyo, Japan). Each male (15–38 days of old for *A. ludens* and 17–27 days old for *A. obliqua*) was placed on ice for 3min. Wild individuals are sexually mature within this age range (Reyes-Hernández and Pérez-Staples, 2017). Males were then placed in a lateral position on a cold gel block and testes were extracted one by one with an insulin syringe (BD Ultra-Fine U-100-0.25mm diameter) for *A. ludens* and a minuten pin (Ento Sphinx 0.20mm diameter) for *A. obliqua*, because it is a smaller species. The tip was bent to form a hook. The syringe or pin was introduced between the last and penultimate abdominal segment. The tip was cleaned with 96% ethanol between each extraction.

In order to corroborate that the testectomy was effective, we verified that there were no sperm in females mated with males without testes. After females had mated with testectomized males and made a choice in the olfactometer tests (see below), they were dissected. The ventral receptacle and the three spermathecae of females mated to testectomized males were dissected. Structures were placed in a drop of saline solution (CS PiSA® solution, Jalisco, Mexico), ruptured and stirred for 1min. We then covered the slide with a cover slip (18mm×18mm). Coverslips were secured with a clear drop of nail polish and left to dry. Subsequently, we assessed the absence of sperm with a phase-contrast microscope (Motic BA310, Hong Kong, China) at a magnification of 100x. We examined the whole sample (324mm^2^). Data from females that contained sperm were omitted. We omitted only two of the 35 examined samples from *A. ludens* and none of the 26 samples from *A. obliqua*.

To corroborate that females had indeed received an ejaculate when mating with testectomized males we compared the size of the MAGs between mated and unmated intact and testectomized males. We allowed females to mate with unmated testectomized or intact males. For *A. ludens* 336 pairs of 15–28days old and for *A. obliqua* 290 pairs of 17–20days of age were observed. Copula duration was recorded. Immediately after mating, the male accessory glands were dissected under a stereomicroscope (Olympus SZX7, Tokyo Japan), photographed, and the area of the two longest arms were measured using Image J (ver. 1.53a) and averaged. Thorax’ length was measured and used as a covariate in the statistical analysis. For *A. ludens* 123 males without testes and 100 males with testes were dissected. For *A. obliqua* 71 males without testes and 91 males with testes were dissected. We then compared the size of MAGs between mated and unmated intact and testectomized males.

#### Mating Observations and Effect of Testectomy on Response to Male Sex Pheromone or Host Volatiles

One day prior to the experiments, we allowed females to copulate with intact or testectomized males. We observed pairs from 17:00 to 22:00h and from 7:00 to 19:00h for *A. ludens* and *A. obliqua*, respectively. We recorded copula duration and latency to mate (time between initial exposure to the female and copulation). Females that copulated were separated for the olfactometer trials. Females and males that did not copulate were discarded. Unmated females were kept in a separate cage.

Observations in the olfactometer consisted of three treatments: unmated females, females mated with intact males with testes (control males), and females mated with males without testes (testectomized males). We followed the same methodology described above. Females chose between two stimuli (pheromone or volatiles). For *A. ludens* we carried out 127 replicates and for *A. obliqua* 87 replicates. We used a total of 381 *A. ludens* and 261 *A. obliqua* females.

To corroborate that the testicular ablation did not affect female response, we also tested olfactory responses in females mated with punctured or intact males. We used the same method as the testectomy, except that males were only punctured without extracting the testes. As a control, *A. ludens* females from 17 to 29days old and *A. obliqua* females from 20 to 27days old were mated with intact males. The day following matings, females were given the choice between male sex pheromone or host volatiles in olfactometer trials. We carried out for *A. ludens* 62 replicates and 50 for *A. obliqua* for a total of 186 *A. ludens* and 150 *A. obliqua*.

### Statistical Analyses

#### Effect of Copulation

The response of unmated or mated females to male sex pheromone, host volatiles, or no stimulus, was analyzed using a Generalized Linear Mixed Models (GLMM) with a binomial response and a logit link function, considering the replicate as a random effect. We only used females that responded to some stimulus in the analyses.

#### Effect of Accessory Gland Products

The male accessory gland size of both testectomized or intact males were each evaluated with analysis of covariance (ANCOVA), where accessory gland size was the dependent variable, type of male was the independent variable and thorax size was the covariate. Latency to mate and copula duration were compared between groups (testectomized males vs. intact males) with a Generalized Linear Models (GLM) with a Gaussian distribution and an identity link function.

To determine the effect of mating with testectomized, intact or no mating on female olfactory response to pheromone or volatiles, we used a GLMM with binomial distribution and a logit link function, followed by a *post-hoc* comparisons test, considering the replicate as a random effect. A Mann Whitney U test for independent samples was used to compare the olfactory response between the two species. A GLMM with a binomial response and a logit link function, considering the replicate as a random effect, was used to compare female response to volatiles or pheromone when mated with punctured or intact males.

All analyses were conducted in Jamovi version 1.0.7.0.

## Results

### Effect of Copulation on Response to Male Sex Pheromone and Host Volatiles

**Figure 1:**
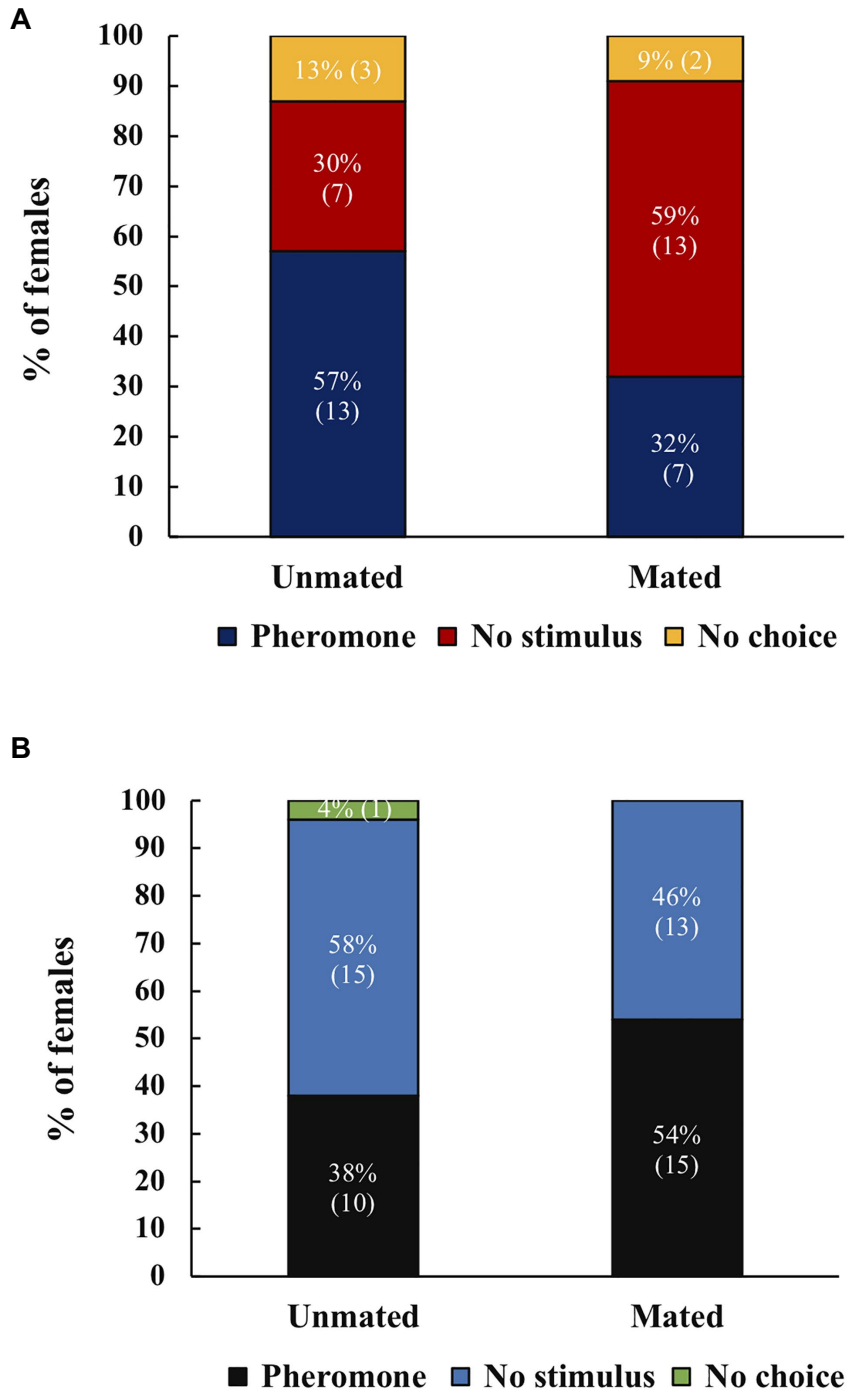
**(A)** Response of unmated or mated *Anastrepha ludens* females to male sex pheromone or no stimulus, (*N*=23 for unmated, *N*=22 for mated). **(B)** Response of unmated or mated *Anastrepha obliqua* females to male sex pheromone or no stimulus, (*N*=26 for unmated, *N*=28 for mated). There were no significant differences. Numbers within parenthesis represent sample size.

When presented with a choice between male pheromone and no stimulus, unmated *A. ludens* females responded more to male pheromone and mated females responded to no stimulus, however this fell short of significance (*χ*^2^=3.49, *df*=1, *p*=0.062, *N*=40; Figure 1A). For *A. obliqua*, mated and unmated females did not show a preference between male pheromone and no stimulus (*χ*^2^=1.09, *df*=1, *p*=0.297, *N*=53; Figure 1B).

**Figure 2:**
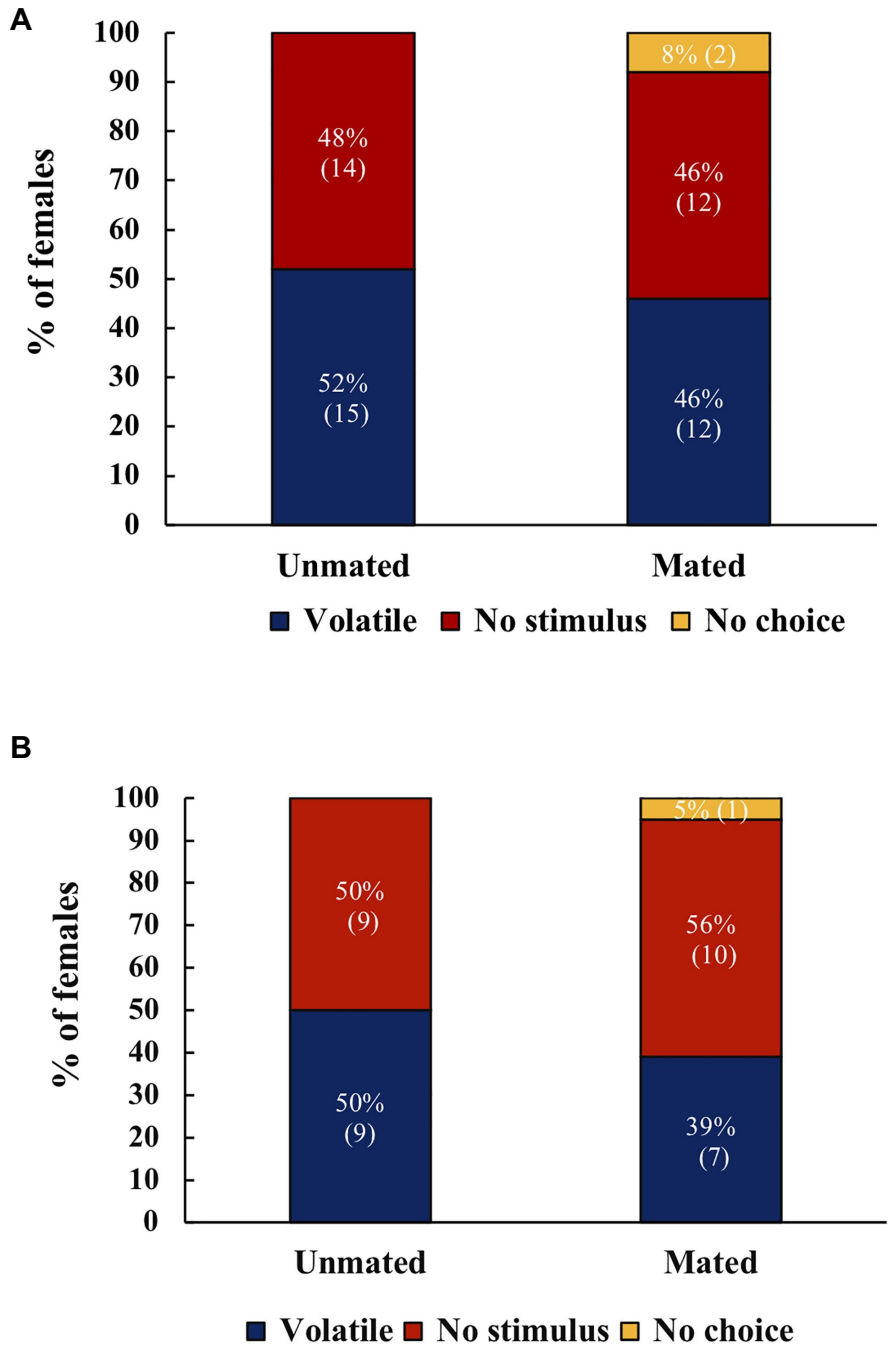
**(A)** Response of unmated or mated *A. ludens* females to host volatiles or no stimulus in the morning (*N*=29 for unmated, *N*=26 for mated). **(B)** Response of unmated or mated *A. ludens* females to host volatiles or no stimulus at dusk (*N*=18 for unmated, *N*=18 for mated). There were no significant differences. Numbers within parenthesis represent sample size.

**Figure 3:**
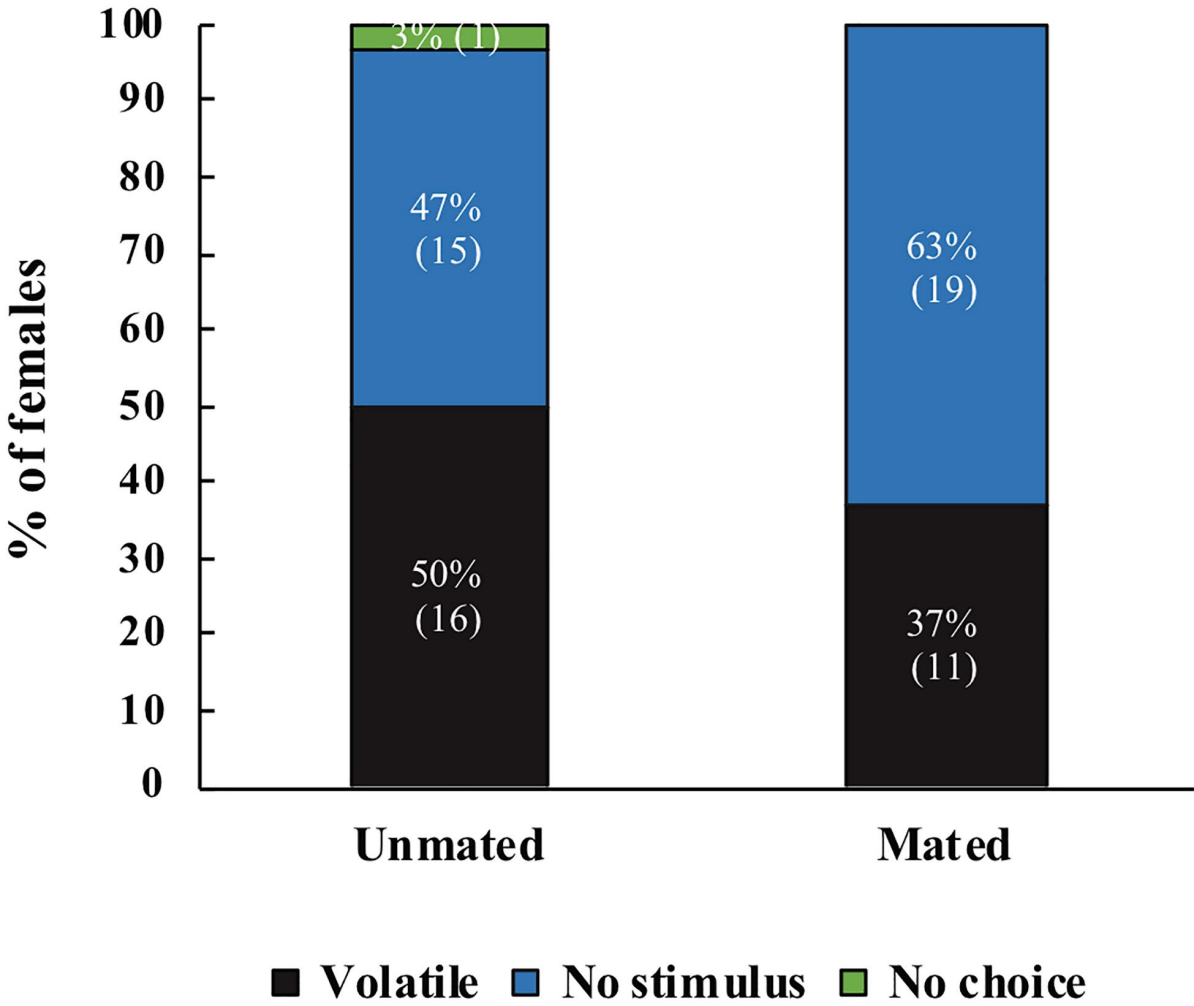
Response of unmated or mated *A. obliqua* females to host volatiles or no stimulus, (*N*=32 for unmated, *N*=30 for mated) There were no significant differences. Numbers within parenthesis represent sample size.

When *A. ludens* females were offered a choice between fruit and no stimulus, there were no significant differences in the response both in the morning (*χ*^2^=0.015, *df*=1, *p*=0.901, *N*=53, Figure 2A), or the evening (*χ*^2^=0.273, *df*=1, *p*=0.601, *N*=35, Figure 2B). For *A. obliqua* there was no significant difference in the response between fruit and no stimulus (*χ*^2^=1.67, *df*=1, *p*=0.197, *N*=61; Figure 3).

**Figure 4:**
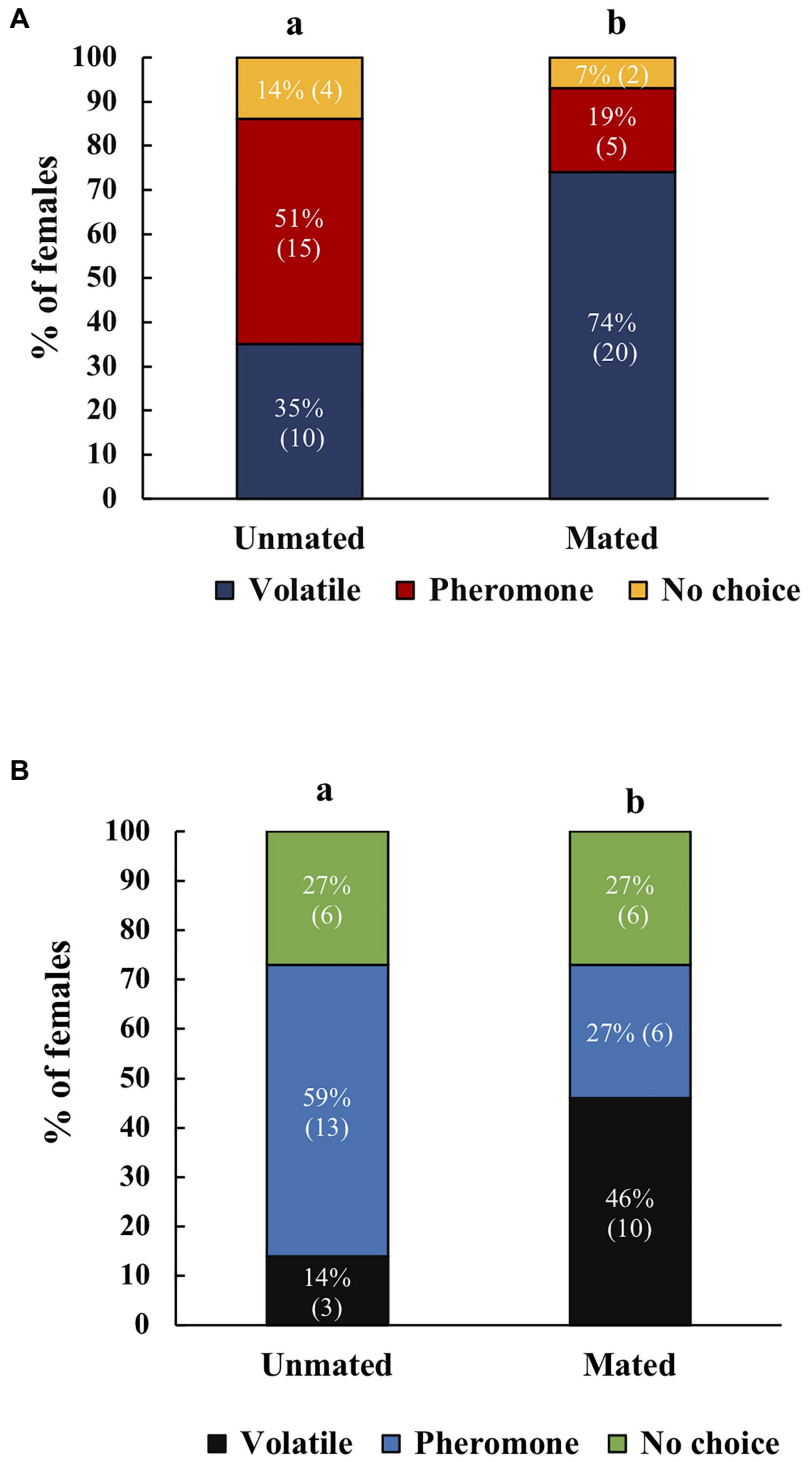
**(A)** Response of unmated or mated *A. ludens* females to host volatiles or male sex pheromone, (*N*=29 for unmated, *N*=27 for mated). **(B)** Response of unmated or mated *A. obliqua* females to host volatiles or male sex pheromone, (*N*=22 for unmated, *N*=22 for mated). The *y*-axis represents the percentage of all females that were tested. Different letters indicate significant differences at the 0.05 level between treatments. Numbers within parenthesis represent sample size.

When females were offered two stimuli (pheromone or volatiles), mated females of both species, responded more frequently to the host, whereas unmated females responded more frequently to the male pheromone (*χ*^2^=7.70, *df*=1, *p*=0.006, *N*=50, *A. ludens*; Figure 4A); (*χ*^2^=5.77, *df*=1, *p*=0.016, *N*=32, *A. obliqua*; Figure 4B).

### Effect of Accessory Gland Products on Response to Male Sex Pheromone and Host Volatiles

We found a significant reduction in accessory gland size after mating, which indicates that males without testes did transfer MAGs. The size of the glands was smaller in mated males than in unmated males both in *A. ludens* without testes (*F*=5.57, *df*=1,120, *p*=0.020, *N*=123), and males with testes (*F*=11.89, *df*=1,97, *p*<0.001, *N*=100). For both models, thorax size had a significant effect on gland size (*F*=7.05, *df*=1,120, *p*=0.009; *F*=8.34, *df*=1, 97, *p*=0.005), for testectomized and intact males, respectively. For *A. obliqua* males without testes, the size of the glands was smaller in mated males compared to unmated males (*F*=5.40, *df*=1,68, *p*=0.023, *N*=71), whereas thorax size was not significantly related to accessory gland size (*F*=2.45, *df*=1,68, *p*=0.122). The male accessory glands for intact males were also smaller for mated than for unmated males (*F*=4.27, *df*=1,88, *p*=0.042, *N*=91), whereas thorax size was not significantly related to accessory gland size (*F*=3.79, *df*=1,88, *p*=0.055; Table 1).

**Table 1:** Accessory gland size (mm^2^) of unmated and mated *A. ludens* and *A. obliqua* males with and without testes.

Species	Males without testes		Males with testes	
	Unmated	Mated	Unmated	Mated
*A. ludens*				
Mean	2.22^a^	2.05^b^	2.27^A^	2.03^B^
Std. error mean	0.052	0.050	0.070	0.047
Sample size	63	60	45	55
*A. obliqua*				
Mean	2.28^a^	2.10^b^	2.32^A^	2.14^B^
Std. error mean	0.046	0.076	0.072	0.054
Sample size	39	32	43	48

Different lowercase letters indicate significant differences between males without tests and uppercase letters indicate significant differences between males with testes.

Males without testes were significantly slower to copulate than males with testes in *A. ludens* (*χ*^2^=26.7, *df*=1, *p*<0.001, *N*=51) and *A. obliqua* (*χ*^2^=23.6, *df*=1, *p*<0.001, *N*=82). There were no significant differences in the duration of copulation between males with and without testes: for *A. ludens* (*χ*^2^=2.39, *df*=1, *p*=0.122, *N*=51) and *A. obliqua* (*χ*^2^=2.54, *df*=1, *p*=0.111, *N*=125).

**Figure 5:**
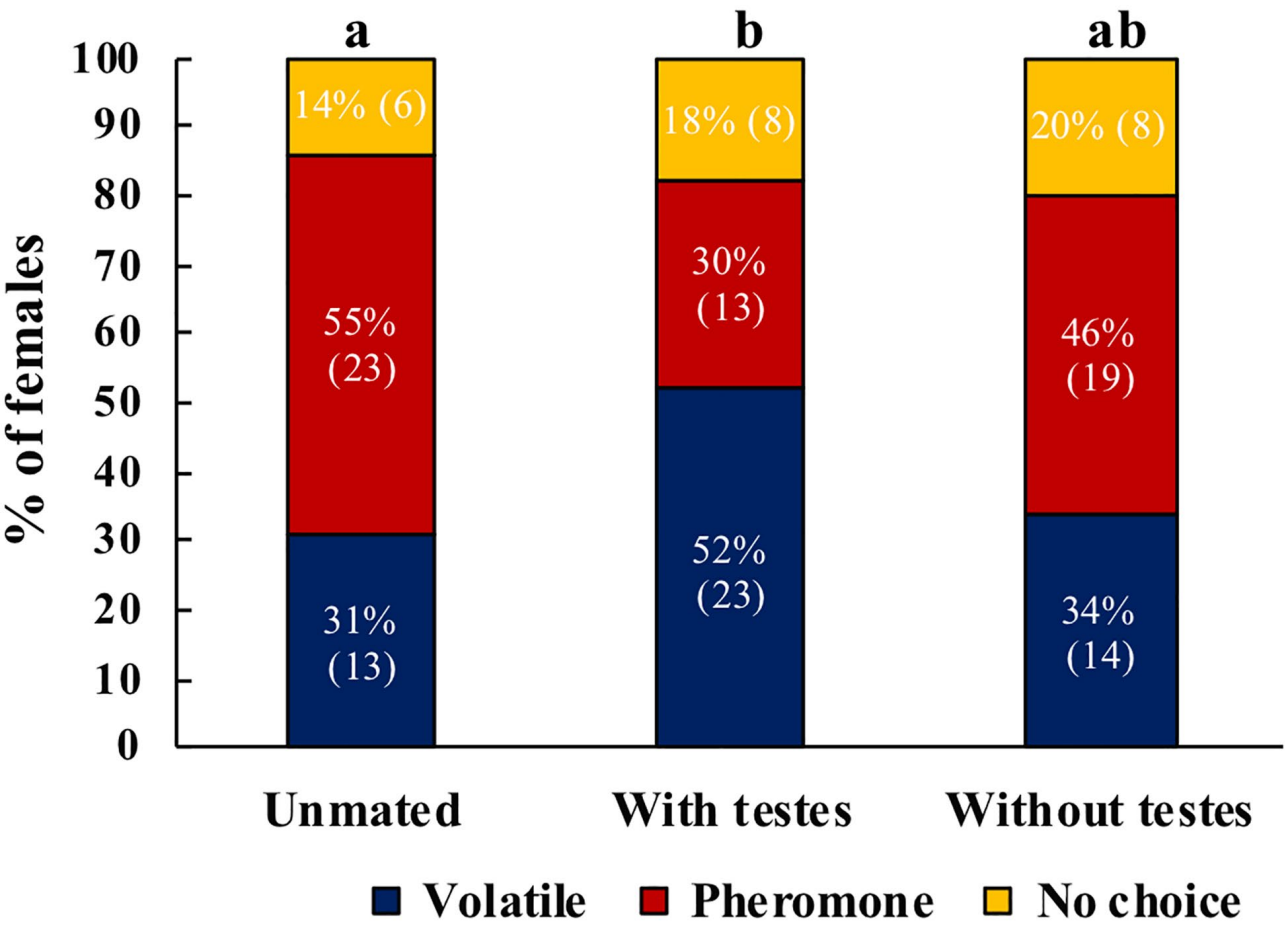
Response of unmated *A. ludens* females (*N*=42), mated with intact males (*N*=44), or mated with testectomized males (*N*=41) to host volatiles or male pheromone. Different letters indicate significant differences between treatments. Numbers within parenthesis represent sample size.

Mated *A. ludens* females were more likely to respond to host volatiles compared to unmated females or to females that only received MAGs (*χ*^2^=5.91, *df*=2, *p*=0.05, *N*=105). *Post-hoc* tests revealed significant differences between unmated and mated females with testes (*z*=2.325, *p*=0.020) and no significant difference between unmated and females mated to testectomized males (*z*=0.536, *p*=0.592; Figure 5). Between treatments, we found that more unmated females responded towards male pheromone, mated females towards volatiles, and no clear preference for females mated with testectomized males (Figure 5).

**Figure 6:**
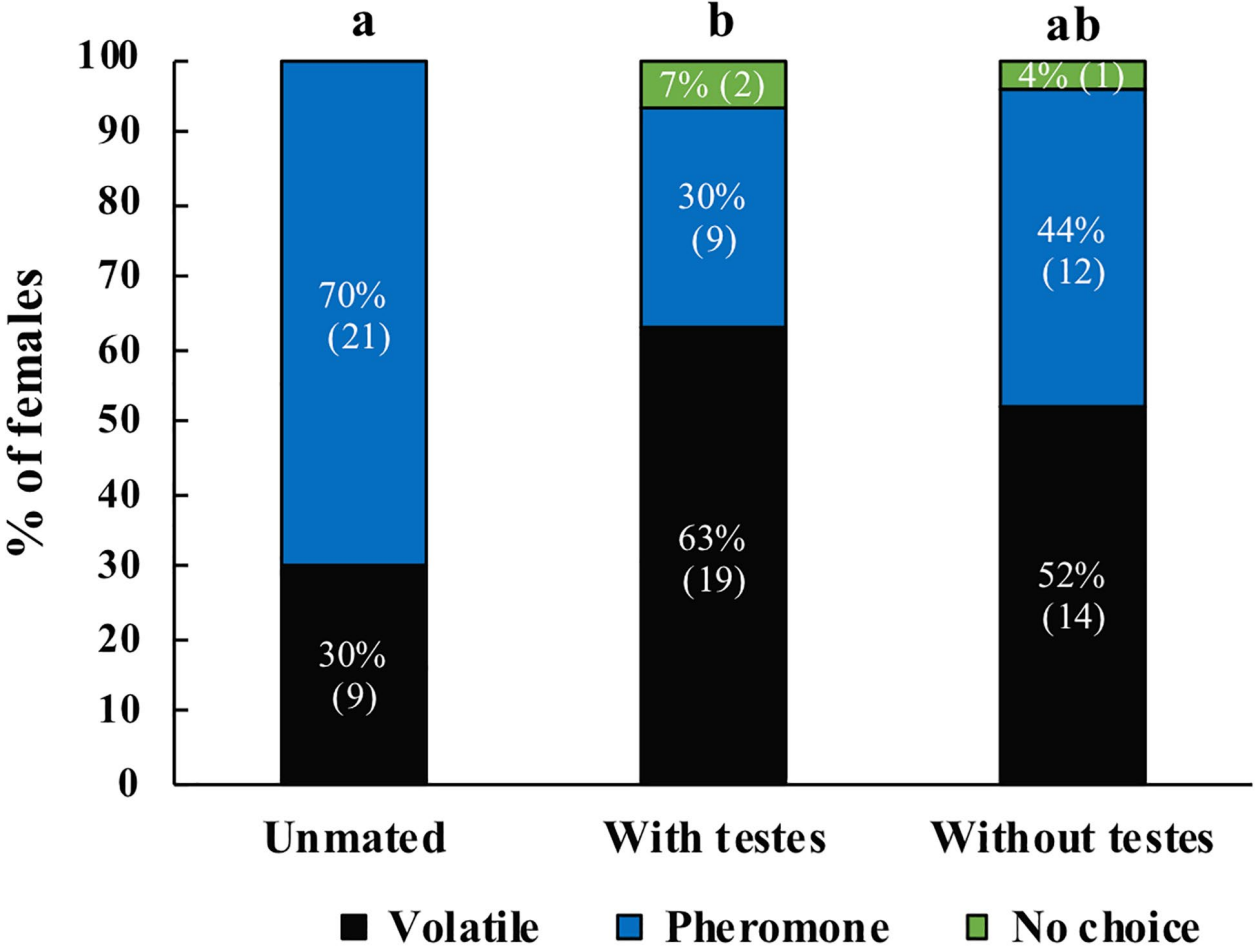
Response of unmated *A. obliqua* females (*N*=30), mated with intact males (*N*=30), or mated with testectomized males (*N*=27) to host volatiles or male pheromone. Different letters indicate significant differences between treatments at the 0.05 level. Numbers within parenthesis represent sample size.

For *A. obliqua* we found the same effect: mated females were more likely to respond to host volatiles compared to unmated females (*χ*^2^=8.08, *df*=2, *p*=0.018, *N*=84). *Post-hoc* tests revealed significant differences between unmated and mated females with testes (*z*=2.81, *p*=0.005), there were no differences between females that only received MAGs compared to unmated (*z*=1.79, *p*=0.074) or females mated with intact males (*z*=1.05, *p*=0.293; Figure 6). Between treatments we found that more unmated females responded towards male pheromone, females mated with intact males responded more towards host volatiles, and no clear preference for females mated with testectomized males (Figure 6). When comparing female response across species we did not find significant differences (Mann Whitney *U*=4,305, *n*_1_=105, *n*_2_=84, *p*=0.747).

As a control, we corroborated that *A. ludens* females mated with punctured males (with testes) responded to host volatiles in the same way as females mated with intact (normal) males (*χ*^2^=1.17, *df*=1, *p*=0.280, *N*=62); 61 and 74% of females responded to host volatiles for females mated with punctured males or with intact males, respectively. For *A. obliqua* there was also no effect of the manipulation on female response (*χ*^2^=0.152, *df*=1, *p*=0.696, *N*=50); 59 and 53% of females responded to host volatiles for females mated with punctured males and intact males, respectively.

## Discussion

Mating elicits changes in female physiology beyond fertilization and copulation. Receiving sperm or MAGs can impact female postcopulatory responses towards further mating or opportunities to oviposit. Here, we determined how copulation and MAGs affect the olfactory response of females in two fruit fly species. We found differential olfactory response in mated compared to unmated *A. ludens* and *A. obliqua* females. Unmated females responded to male pheromones over oviposition host volatiles, whereas mated females responded to oviposition host volatiles over male pheromones. On the other hand, females of both *A. ludens* and *A. obliqua* that only received MAGs during copulation did not demonstrate this response for host volatiles.

Unmated and mated females can respond differently to certain stimuli; thus, we expected mated females to respond to host volatiles as copulation could trigger a series of reactions in the central nervous system or directly in effector organs (muscles; Kingan et al., 1993), which would not occur in unmated females. Our results are in agreement with the response of unmated *A. ludens* and *Bactrocera tryoni* (Froggatt) females that prefer male sex pheromone, while copulation causes a reduction in the reaction to this pheromone (Robacker et al., 1985; Robacker and Garcia, 1990; Devescovi et al., 2021). Previous studies with *A. obliqua* and *Anastrepha striata* (Schiner) have shown that the physiological state (unmated or mated) of females did not affect their attraction to different fruit volatiles (Diaz-Santiz et al., 2015; López-Ley et al., 2016). However, here we show that when given a choice between host volatiles and pheromone, mated females clearly responded to host volatiles.

Both species responded similarly to *C. capitata*, where there is a change in olfactory response after copulation with mated females preferring host volatiles over male pheromone (Jang, 1995; Jang et al., 1999). However, in contrast to what was found in *C. capitata* with male MAG injections, we did not find that the response for host volatiles was induced by receiving MAGs. In our study, neither *Anastrepha* species showed a change in behavior when only receiving MAGs. Females mated with testectomized males did not behave either as unmated or mated females: their response was intermediate between the two. This leads to the question of what the functions of MAGs in these two species are. In *A. ludens*, MAGs injection into females does not decrease female receptivity, influence ovary development, or increase egg number (Abraham et al., 2014, 2016; Reyes-Hernández et al., 2021), however, the females do require MAGs and sperm for the decrease in receptivity in this species (Abraham et al., 2014, 2016). In other Diptera, MAGs can have a number of effects including influencing female longevity (Chapman et al., 1995; Villarreal et al., 2018), increasing oviposition (Kalb et al., 1993) and decreasing remating (Chen et al., 1988; Radhakrishnan and Taylor, 2007). Even within *Anastrepha* there is variation in the impact of MAGs; in *Anastrepha suspensa* (Loew) and *A. ludens* they do not inhibit female receptivity or induce oviposition (Lentz et al., 2009; Abraham et al., 2016), while in *Anastrepha fraterculus* (Wiedemann) they do reduce female sexual receptivity (Abraham et al., 2012). In *A. fraterculus*, when MAGs are injected into females, there is inhibition of receptivity, but this is not of the same magnitude as that produced by normal copulation, where all the ejaculate is transferred (Abraham et al., 2011). Our study also suggests that it is the complete ejaculate that produces the post-copulatory olfactory changes in females.

In *A. ludens*, transcriptomic studies have shown important changes after mating and MAG-injection suggesting that MAGs could be performing functions such as increasing life span in response to stressful environmental factors, (e.g., low humidity or nutrient limitation), as well as dietary detoxification, glucose regulation, and increasing egg production through translation stimulation (Sirot et al., 2021). In *A. obliqua*, MAG function is unknown, but, based on the results of our study, they do not seem to have an effect on olfactory response.

Mating with testectomized males induced no olfactory response switch relative to females mated with intact males. Based on these results, we can infer that in these two tephritid species: (1) the mating-induced change in olfactory response could be caused by sperm and not by MAGs, since females that only received MAGs did not show a response for fruit volatiles compared to those that received a complete ejaculate, or (2) females need both MAGs and sperm in order to display a behavioral olfactory switch to fruit volatiles. However, based on other phenotypes studied in *Anastrepha* spp. there is more evidence to support the latter explanation. For example, in *A. ludens*, female receptivity is reduced only when they receive a full ejaculate with both sperm and MAGs (Abraham et al., 2016). In *A. fraterculus*, females must receive both sperm and MAGs to have a normal post-copulatory fecundity boost (Abraham et al., 2020). Future studies should evaluate whether females mated with both sperm-and MAG-depleted males have different olfactory responses compared to females only receiving MAGs, and whether the effects of MAGs become more evident on a longer time scale.

Finally, it is important to note that the methodology of testicular ablation had not been used previously in tephritids, even though it has been used in other insect species such as *Heliothis zea* (Boddie) (Raina, 1989) and *Gryllus texensis* Cade and Otte (Worthington et al., 2015). In order to determine how copulation and MAGs change female postcopulatory behavior, other techniques have been used in tephritids, such as sperm depletion or depletion or injections of MAGs (Kuba and Itô, 1993; Jang, 1995; Lentz et al., 2009; Abraham et al., 2020). The advantages of using testectomized males over these other methods are the specific and natural transfer of MAGs, allowing the female to have an actual mating receiving all associated stimuli and ejaculate components except sperm, and that females are not punctured and thus are not harmed. Our study employing testectomy in tephritids broadens the possibilities of performing a range of studies for investigating the function of MAGs in other Diptera.

Mated females had a greater response to host volatiles than to the male’s sexual pheromone. In the context of behavioral response to traps, this would imply that after mating, females would search for a host and would not be attracted to pheromone based baits. Unfortunately, the baits used in the field to attract wild females are food lures that mostly attract unmated females (Weldon et al., 2008; Epsky et al., 2014; Perea-Castellanos et al., 2015), or male lures where only unmated and not mated females respond (Fitt, 1981). These results reinforce the need for baits based on host volatiles to attract mated females. This population should be targeted as mated females lay more eggs than unmated females and only eggs laid by mated females will develop into larvae which damage fruit and increase population size (Reyes-Hernández et al., 2021). We conclude that copulation does change female behavior in the olfactory response to host volatiles and male sex pheromone in both species, while MAGs alone do not elicit this response.

## Data Availability Statement

The raw data supporting the conclusions of this article will be made available by the authors, without undue reservation.

## Author Contributions

GC-G: conceptualization, formal analysis, investigation, methodology, project administration, writing-original draft, and writing review and editing. LS: conceptualization, methodology, writing review and editing, and formal analysis. SA and NF-E: writing review and editing and supervision. FD-F: writing review and editing, supervision, and formal analysis. ML-O: funding acquisition, resources, and writing review and editing. DP-S: conceptualization, supervision, project administration, methodology, validation, writing review and editing, and writing-original draft. All authors contributed to the article and approved the submitted version.

## Funding

GC-G was funded by CONACyT for a doctoral scholarship (no. 203190).

## Conflict of Interest

The authors declare that the research was conducted in the absence of any commercial or financial relationships that could be construed as a potential conflict of interest.

## Publisher’s Note

All claims expressed in this article are solely those of the authors and do not necessarily represent those of their affiliated organizations, or those of the publisher, the editors and the reviewers. Any product that may be evaluated in this article, or claim that may be made by its manufacturer, is not guaranteed or endorsed by the publisher.
